# Apple Leaf Diseases Recognition Based on An Improved Convolutional Neural Network

**DOI:** 10.3390/s20123535

**Published:** 2020-06-22

**Authors:** Qian Yan, Baohua Yang, Wenyan Wang, Bing Wang, Peng Chen, Jun Zhang

**Affiliations:** 1 School of Electrical and Information Engineering, Anhui University of Technology, Ma’anshan 243032, China; yanqian201288@163.com (Q.Y.); wywangahut@163.com (W.W.); 2 Key Laboratory of Power Electronics and Motion Control Anhui Education Department, Anhui University of Technology, Ma’anshan 243032, China; 3 School of Information and Computer, Anhui Agricultural University, Hefei 230036, China; ybh@ahau.edu.cn; 4 Co-Innovation Center for Information Supply & Assurance Technology, Anhui University, Hefei 230032, China; pchen@ahu.edu.cn (P.C.); junzhang@ahu.edu.cn (J.Z.)

**Keywords:** apple leaf diseases, transfer learning, deep learning, convolutional neural networks

## Abstract

Scab, frogeye spot, and cedar rust are three common types of apple leaf diseases, and the rapid diagnosis and accurate identification of them play an important role in the development of apple production. In this work, an improved model based on VGG16 is proposed to identify apple leaf diseases, in which the global average poling layer is used to replace the fully connected layer to reduce the parameters and a batch normalization layer is added to improve the convergence speed. A transfer learning strategy is used to avoid a long training time. The experimental results show that the overall accuracy of apple leaf classification based on the proposed model can reach 99.01%. Compared with the classical VGG16, the model parameters are reduced by 89%, the recognition accuracy is improved by 6.3%, and the training time is reduced to 0.56% of that of the original model. Therefore, the deep convolutional neural network model proposed in this work provides a better solution for the identification of apple leaf diseases with higher accuracy and a faster convergence speed.

## 1. Introduction

Leaf diseases are one of the main obstacles to apple production. Among them, scab, frogeye spot, and cedar rust are three most common types of apple leaf diseases and have a bad impact on apple growing. Therefore, the detection of apple leaf diseases has attracted more and more attention, and the early identification of apple leaf disease is very important for the intervention of treatment. In the past, disease identification methods were generally divided into manual identification and an expert system. However, both of them are highly dependent on fruit growers and experts and are time-consuming and usually poor in generalization.

With the development of machine learning methods, some computational models have been proposed for plant disease diagnosis based on different algorithms. Some studies have found diseased regions by K-means clustering-based segmentation and build disease recognition models using supervised learning methods, including the random forest, support vector machine (SVM), and K-nearest neighbor methods [1,2,3]. Rothe et al. used an active contour model for image segmentation and extracted Hu’s moments as features for the training of an adaptive neuro-fuzzy inference system, by which a classification accuracy of 85% can be achieved [4]. Gupta et al. proposed an autonomously modified SVM-CS model where a SVM model was trained and optimized using the concept of a cuckoo search [5]. However, these classification features are heavily depended on man-made selection and the recognition rates are not satisfactory.

In recent years, convolutional neural networks (CNNs) have shown good results in recognition tasks by reducing the need for image preprocessing and improving the identification accuracy [6,7,8,9,10,11,12,13]. Leaf disease recognition based on CNNs has become a new hotspot in the agricultural informatization area [14,15,16]. Lu et al. proposed a rice disease identification method based on deep CNN techniques and achieved an accuracy of 95.48% on a dataset of 500 natural images of diseased and healthy rice leaves [17]. Zhang et al. proposed the improved GoogLeNet and Cifar10 models and obtained the average identification accuracies of 98.9% and 98.8%, respectively [18]. Liu et al. designed a novel architecture of AlexNet to detect apple leaf diseases, and the experimental results showed that this approach achieved an overall accuracy of 97.62% for disease identification [19]. Although the recognition accuracy of these CNN models is higher than that of traditional machine learning methods, there are still some shortcomings—such as high model complexity, much more parameters, and a long training time—which prevent their application in real environments.

In this work, we propose a method for apple leaf disease identification based on an improved deep convolution neural network architecture which can effectively reduce the model complexity and training time. The network proposed in this work adopts the concept of transfer learning to pre-train a VGG16 network and adjusts the network structure by removing three fully connected layers, adding a global average pooling layer, a batch normalization layer, and a fully connected layer. Based on a benchmark dataset, the proposed model, which can reach a 89% reduction in the model parameters of the original VGG16 model, greatly reduced the training time and achieved a higher accuracy rate.

## 2. Methods

### 2.1. Data

**Figure 1:**
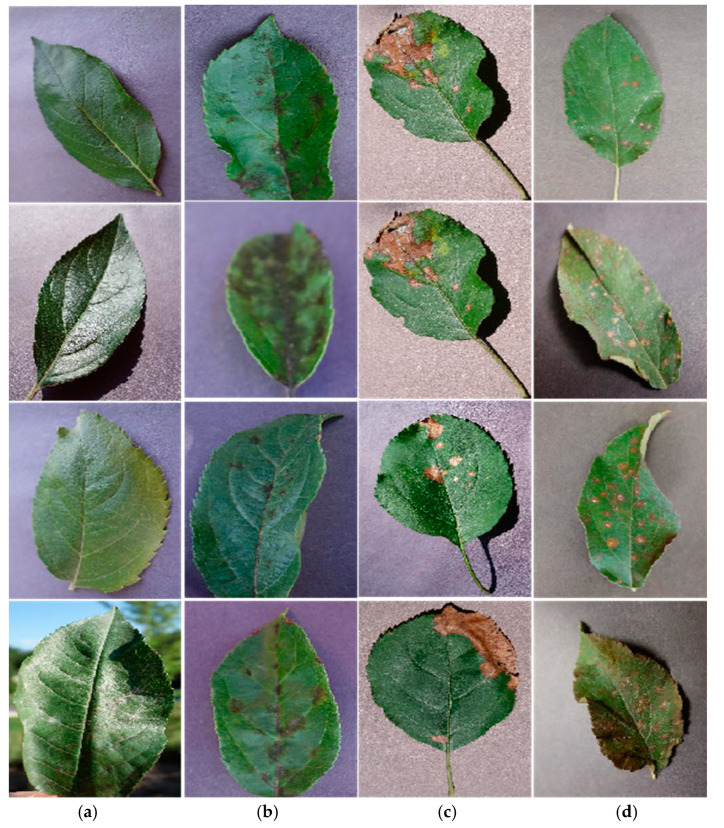
Four kinds of apple leaves. (**a**) Healthy, (**b**) scab, (**c**) frogeye spot, (**d**) cedar rust.

The dataset in this work is from the “2008 ‘AI Challenger’ Global Challenge” and includes 10 kinds of plants with 27 categories of diseases. This work addresses the automatic identification of apple leaf diseases, therefore only apple leaves are selected from this dataset. There are four categories of apple leaf images within the dataset, and Figure 1 lists some of them. With the exception of healthy leaves, three types of disease images—i.e., scab, frogeye spot, and cedar rust—are collected within the dataset. Typically, the lesions on scab leaves are gray-brown and nearly round or radial, frogeye spot is tan and the shape is flakes or dots, and cedar rust leaves have round orange-yellow lesions with red edges. Some spot and cedar rust lesions are similar in color and shape, which increases the difficulty in recognition by computational methods.

In this work, there are 2446 pictures collected within our dataset, where 1340 of them are healthy, 411 are scab, 487 are frogeye spot, and 208 are cedar rust. In the original dataset, the dataset was divided into two subsets—i.e., 2141 pictures were for model training and the remaining 305 ones for testing. The details about the dataset are shown in Table 1.

### 2.2. VGG16 and Transfer Learning

#### 2.2.1. VGG16

With the rapid development of deep learning, CNNs had been applied widely in different fields, especially in image classification and recognition and target location and detection [20]. A CNN is a special multi-layer perceptron (MLP) or multilayered feed forward neural network, which generally consists of an input layer, convolution layer, pooling layer, fully connected layer, and output layer. The convolution layer can realize dimensionality reduction and feature extraction by implementing two design concepts: local perception and parameter sharing. The pooling layer can reduce the size of the data, where smart sampling also has the invariance of local linear transformation, which enhances the generalization ability of convolutional neural networks. The fully connected layer acts as a classifier in the whole neural network. It is common for multiple fully connected layers to be used after several rounds of convolution, and the resulting structure of the last convolutional layer is flattened [21,22].

**Figure 2:**
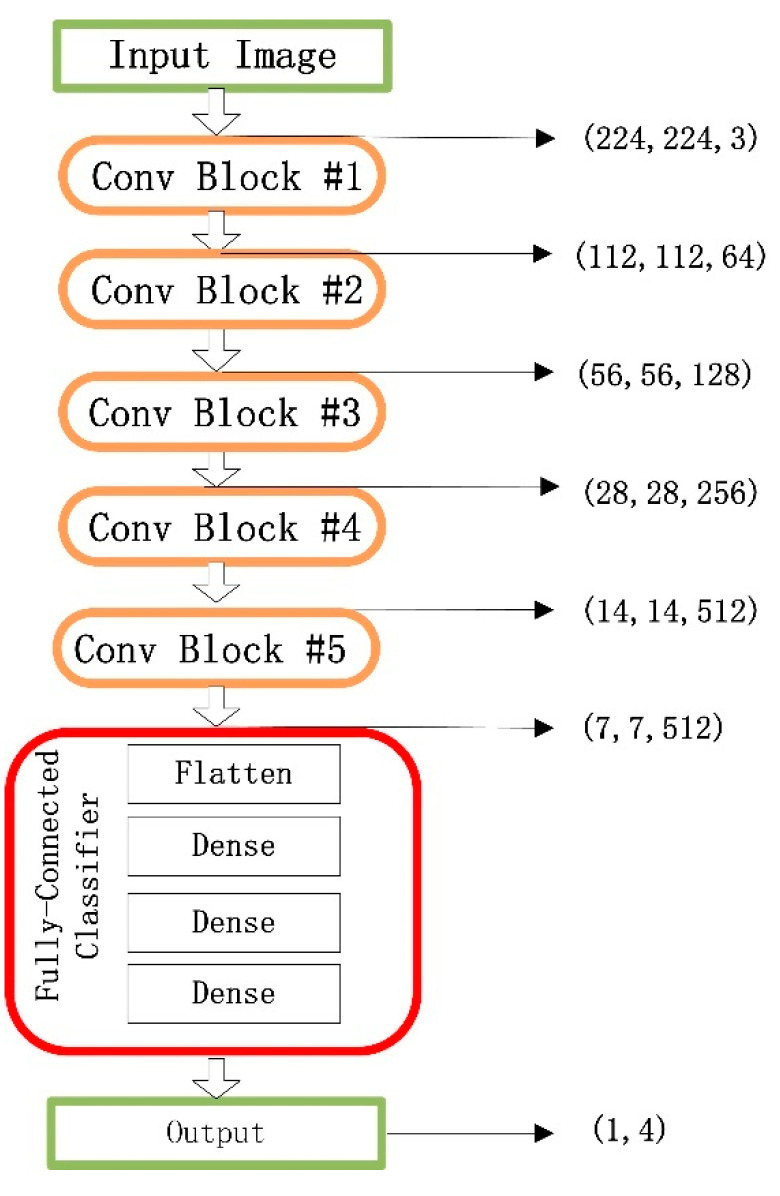
A schematic of the VGG16 architecture.

The VGG16 contains 16 convolutional layers with very small receptive fields, 3 × 3, and five max-pooling layers of size 2 × 2 for carrying out spatial pooling, followed by three fully connected layers. A classical VGG16 model involves 144 million parameters, where rectification nonlinearity (ReLU) activation is applied to all hidden space pooling and the softmax function is applied in the final layer [23]. The model also uses dropout regularization in the fully connected layers. A schematic of the VGG16 architecture is shown in Figure 2, where the marked red box shows a classifier consisting of three fully connected layers.

#### 2.2.2. Transfer Learning

CNNs typically require a large annotated image dataset to achieve a high predictive accuracy. However, the acquisition of such data is difficult and labeling them is costly in many areas. In light of these challenges, the concept of transfer learning is adopted in many previous studies for solving cross-domain image classification problems and has been shown to be very useful, where the “off-the-shelf” features of well-established CNNs, such as VGG16, AlexNet, and GoogLeNet, are pre-trained on large-scale annotated natural image datasets, such as ImageNet, where 15 million images are involved [24,25,26,27].

One common strategy of transfer learning is feature transfer, which removes the last layer of the pre-trained network and sends its previous activation values, which can be regarded as feature vectors, into classifiers for training. Another is parameter transfer, which only needs to re-initialize a few layers of the network, such as the last layer, and the other layers directly using the weight parameters of the pre-trained network, while a new dataset is used to finetune the network parameters [28,29,30]. 

Because of the small amount of data in this work, training a neural network from scratch will take a long time, and the data insufficiency easily causes an over-fitting problem, which will bring the model poor robustness. Therefore, we can use the idea of transfer learning, where a pre-trained model is built on ImageNet to optimize the classification and recognition of apple leaf diseases. Herein, the VGG16 is fine tuned to fit our own data, which can save a lot of training time.

### 2.3. Improved CNNs Based on VGG16

A classical VGG16 network has a strong ability of image feature extraction and recognition. Its core idea is to use smaller convolution kernels to increase the depth of the network, which was the key to win the runner-up position in positioning and classification tasks in the ILSVRC Challenge in 2014. However, the VGG16 model has a huge amount of parameters, which will cause a slow convergence speed, long training time, and large storage capacity in practical applications.

**Figure 3:**
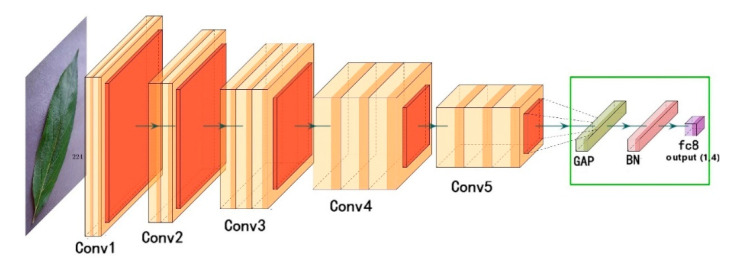
A schematic of the proposed CNN based on VGG16.

To address these problems, this work improves the VGG16 model by using a global average pooling layer, a batch normalization layer and a fully connected layer to replace the three fully connected layers in the original model. The global average pooling layer is used to replace the fully connected layer to reduce the parameters, and the batch normalization layer is added to improve the convergence speed. In order to avoid a long training time, the weights of the convolution layers are pre-trained by VGG16 on ImageNet. The stochastic gradient descent (SGD) optimizer is replaced by an adaptive moment estimation (Adam) to accelerate the convergence of the network. The network structure is shown in Figure 3, where the improvement of a classifier consisting of a global average pooling layer, a batch normalization layer, and a fully connected layer is shown within the marked green box.

#### 2.3.1. Global Average Pooling Layer (GAP)

**Figure 4:**
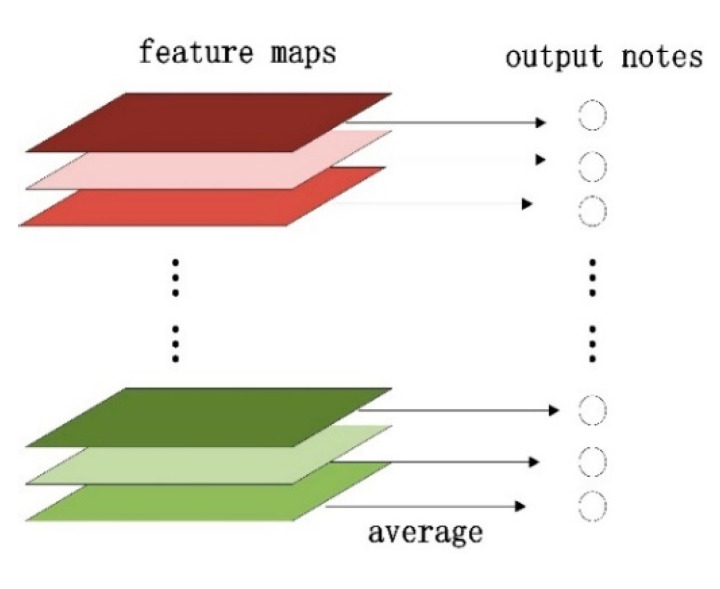
Global average pooling.

Global average pooling is to regularize the whole network structure to prevent over-fitting and reduce the dimensions from 3D to 1D [31,32]. In this work, the feature maps in the last convolution layer are averaged into a series of 1D outputs which is shown in Figure 4. A GAP can omit the expansion of the feature maps into vectors and full connection processing, and therefore greatly reduces the number of parameters. The advantage of a GAP over a fully connected layer is that it can preserve the convolution structure better by enhancing the correspondence between the feature maps and analogy, making the classification of the feature map credible and well-explained.

#### 2.3.2. Batch Normalization (BN)

In deep learning, because the number of layers in the network is very large, if the data distribution at a certain layer starts to deviate significantly, this problem will intensify as the network deepens, which will increase the difficulty of the model optimization. Therefore, normalization helps to alleviate this problem. This method of batch normalization divides the data into several groups and updates the parameters according to the groups. The data in one group jointly determines the direction of the gradient and reduces the randomness when declining. On the other hand, because the number of samples in the batch is much smaller than the entire dataset, the amount of calculation has also dropped significantly. The batch normalization layer normalizes the inputs to the layer before the activation function is implemented, which can solve the problems of input data offset and increase [33].

Based on the BN algorithm, the parameters of the input layer are normalized and the activation function cannot affect the distribution of neurons. The importance of neurons will be weakened and some of them may be removed automatically. Because of the normalization of each epoch, the risk of parameter changes caused by a different data distribution is reduced and the convergence speed is accelerated.

#### 2.3.3. Adaptive Moment Estimation

Adam is an extension of the stochastic gradient descent algorithm which can iteratively update the neural network weights based on training data [34,35]. This method not only stores the exponential decay mean of the square gradient but also preserves the exponential decay mean of the previously calculated first-order and second-order moment estimation of the gradient. It also designs different adaptive learning rates for different parameters. Optimization algorithms such as SGD maintain a single learning rate during the training process, and Adam can iteratively update the neural network weights based on the training data. When the parameters are backpropagated and updated, the Adam algorithm can better adjust the learning rate. Thus, Adam has a fast convergence speed and effective learning effect. It can also correct the problems existing in other optimization techniques, such as the loss function fluctuation caused by the disappearance of the learning rate, slow convergence, or parameter updating with high variance.

## 3. Results and Discussion

In this work, the proposed model was implemented with the Keras deep learning framework using a Intel^®^ Core™ i7-8750H GPU (LENOVO, Jiangsu, China). The ImageNet pre-trained VGG16 CNN implemented within Keras Applications takes in a default image input size of 227 × 227. Therefore, all the pictures in our dataset were cut to the same size of 227 × 227.

The proposed CNN is trained on 2141 training pictures and tested on 305 ones, and the confusion matrices of the prediction results is shown Table 2. It can be found that the cedar rust classification is totally accurate, only one healthy picture is misclassified as scab, and only one is misclassified as healthy in both of scab and frogeye spot categories. 

**Figure 5:**
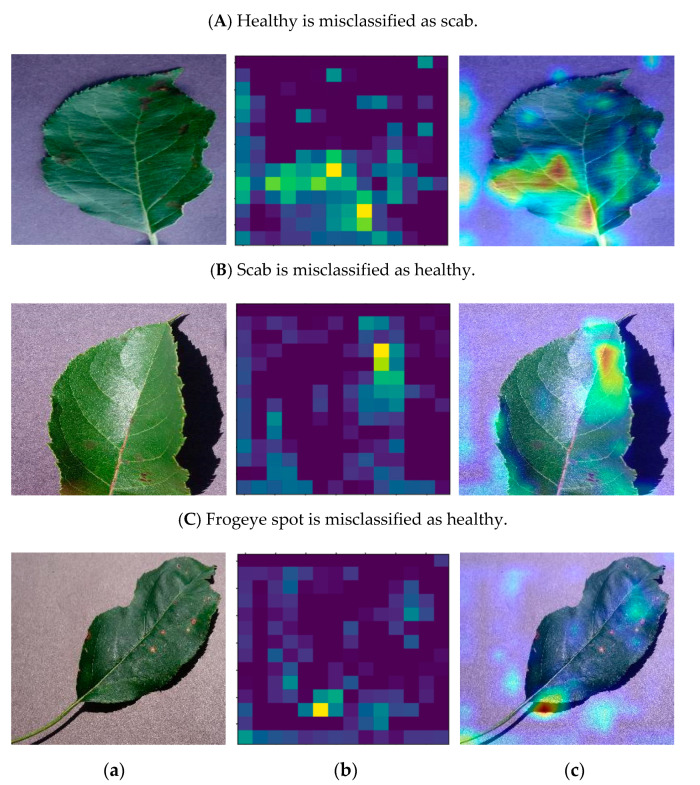
The details of three misclassified pictures: (**a**) the original picture, (**b**) the visualization of the final convolution layer, (**c**) the superposition of the heat map of the original picture.

For the three misclassified pictures in the original dataset, Figure 5 lists the original one, its visualization of the last convolution layer and the superposition of the heat map of the original picture. There are some enlightenments can be found from these pictures. In Figure 5b, the strong light and small disease features may lead to the inaccurate extraction of disease features by the model. The frogeye spots in Figure 5c are small in size and light in color, which will leads to prediction errors with comparison to the dark area, for light is strongly learned in the network and therefore has a bigger weight.

### 3.1. Comparison of Model Performance

**Figure 6:**
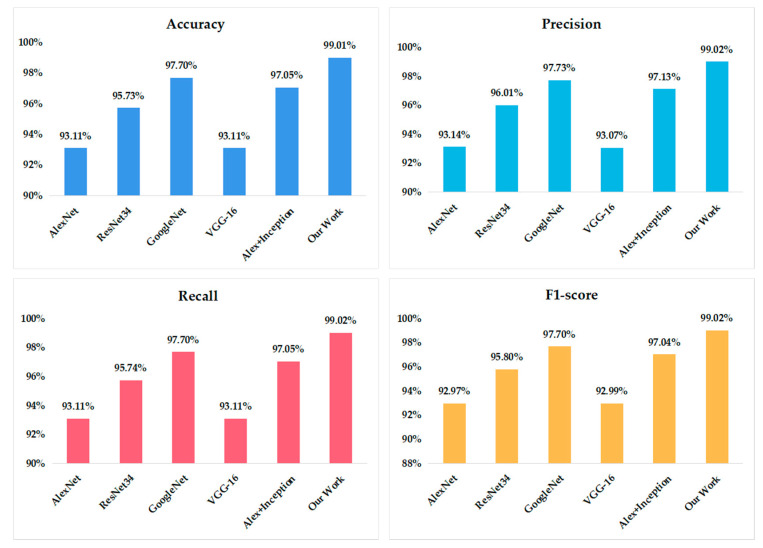
Recognition accuracy of different models.

To evaluate the performance of the proposed VGG model, four typical convolutional neural networks—i.e., AlexNet, GoogleNet, Resnet-34, and VGG16—are also implemented. Another apple leaf disease recognition structure presented by Liu et al., where the inception structure was added into the AlexNet framework, has also been compared. The recognition accuracy of the different models is shown in Figure 6.

It can be found that the accuracy of AlexNet and the original VGG16 is 93.11%, ResNet34 is 95.73%, and GoogleNet can reach 97.70%. When the inception structure was combined with AlexNet, the identification accuracy can be increased to 97.05%, which is higher than the original AlexNet. It can be seen that our work achieves the highest accuracy in the identification of apple leaf diseases—i.e., a 99.01% accuracy—which demonstrates the effectiveness of the proposed model. Compared to the other five models, whether in terms of precision, recall, or F1-score, our model achieved the highest value.

Table 3 shows the precision, recall, f1-score, and accuracy of different models achieved for the four categories of apple images. The Table 4 shows that AlexNet does not learn the features of the scab well enough, and the detection effect is poor; the improved Alex + Inception model recognition is better than the original Alex; what is more, the original VGG16 network has the worst learning of each feature. For these four-leaf types, all the networks have the best recognition rate for healthy and the lowest scab recognition rate. Regardless of the accuracy or the detection index of each leaf type, our model achieved the best results. In general, our model has the best recognition effect.

### 3.2. Convergence Rate Analysis

**Figure 7:**
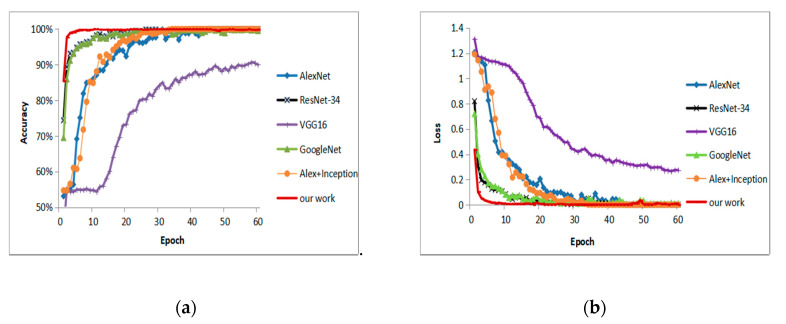
Convergence comparison: (**a**) accuracy values; (**b**) loss values.

The loss values in this work are calculated by cross entropy. Figure 7 shows the accuracy and loss values of the five models during training. The experimental results show that AlexNet, ResNet-34, GoogleNet, Alex + Inception, and our convolutional neural network converge within 60 training epochs, while VGG16 converges slowly. It can be found the proposed network structure converges in 10 training epochs, which is faster than the other five CNN models. The training process of GoogleNet is similar to the process of ResNet-34, and both converge after 20 training epochs, and AlexNet and the Alex + Inception model tend to be stable after 40 epochs.

### 3.3. Training Time and Parameters

Table 4 shows the number of parameters for each model and training time required when the model becomes stable. It can be found that the classical VGG16 model has the most parameters and the longest training time, the Alex + Inception model has the least training parameters, and AlexNet has the shortest training time. Our improved model can reduce 119,534,592 training parameters in comparison to the original VGG16 model. The convolutional neural network proposed in this work has fewer training parameters than AlexNet, ResNet34, and VGG16. The training time of the proposed model is 692 s, which is similar to that of ResNet34 and GoogleNet.

### 3.4. Comparison of Optimal Algorithms

**Figure 8:**
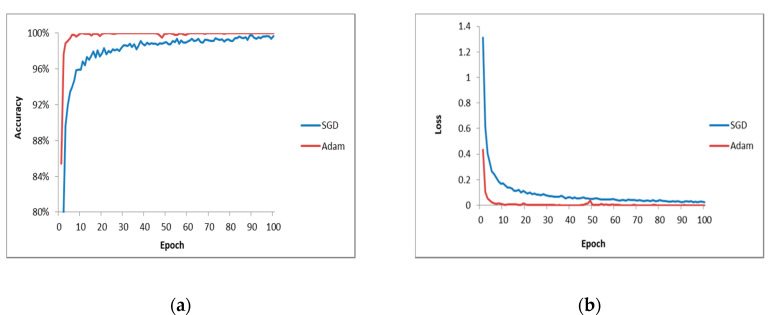
Comparison of the optimal algorithms: (**a**) accuracy values; (**b**) loss values.

The optimization algorithm is of great importance for the model performance. In this work, the SGD optimization algorithm in the original VGG16 is replaced by the Adam optimization algorithm to improve the converge rate. Figure 8 shows the training process of these two optimization algorithms with same learning rate of 1 × 10^−5^. The results show that the model using the Adam algorithm has a faster convergence speed. It can be found that the accuracy of testing is 98.03% when the SGD algorithm is used, while that of the Adam algorithm is 99.01%. From the loss curve in Figure 8, it can be seen that the Adam algorithm can converge quickly and is more stable than SGD.

### 3.5. Data Augmentation

In this work, the dataset used herein includes only 2446 pictures, which is very small in comparison to that with which the VGG16 was pre-trained. In order to evaluate the performance of the proposed method, a data augmentation strategy is adopted to amplify the original dataset and test the classification performance on it. The augmented dataset is generated based on the original dataset by image geometric transformation, color changing, and noise adding, which increase the size of the test dataset from 2141 to 21,410.

**Figure 9:**
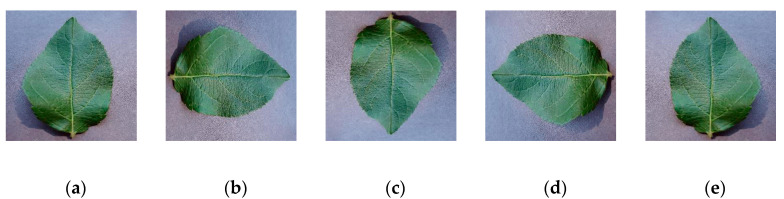
Direction disturbance: (**a**) initial, (**b**) 90, (**c**) 180, (**d**) 270, (**e**) horizontal flip.

Image rotation and flipping are two types of image geometric transformations where only the location of each pixel is changed. Rotating the pictures at different angles and flipping can expand the diversity of directions. It is generally difficult to capture each picture from different directions, and therefore to simulate this situation to eliminate the effect of direction on picture recognition, we rotated the original image around the center point by 90, 180, and 270 and when flipped horizontally. As shown in Figure 9, after rotation and flipping, the number of pictures increased by 4 times the original data set.

**Figure 10:**
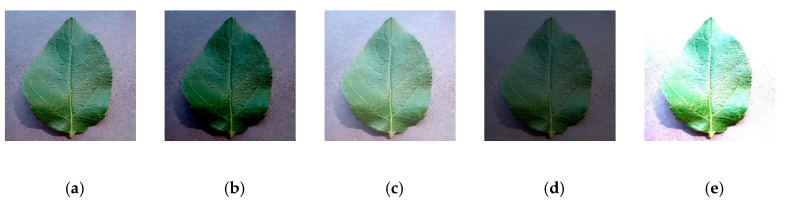
Color enhancement: (**a**) initial, (**b**) low brightness, (**c**) high brightness, (**d**) low contrast, (**e**) high contrast.

Adjusting the brightness, contrast, and hue of the image is another common image augmentation method widely used in image processing. During the process of image acquisition, pictures may be affected by different weather and exposed to different intensities of light, which possibly affects the experimental results. In order to simulate image collection under different light backgrounds, we adjusted the brightness and contrast, as shown in Figure 10, and the data was expanded by 4 times.

**Figure 11:**
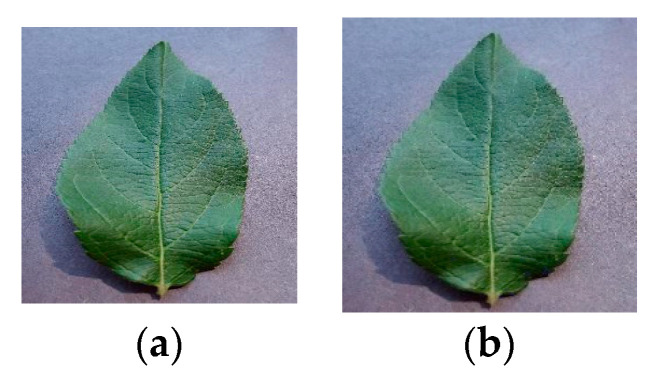
Gaussian noise: (**a**) initial, (**b**) Gaussian noise.

To gain some insight into the noise effect on apple leaf pictures, which is also a common factor come from image acquisition equipment and the natural environmental, we added Gaussian noise to the original image, which is shown in Figure 11.

**Figure 12:**
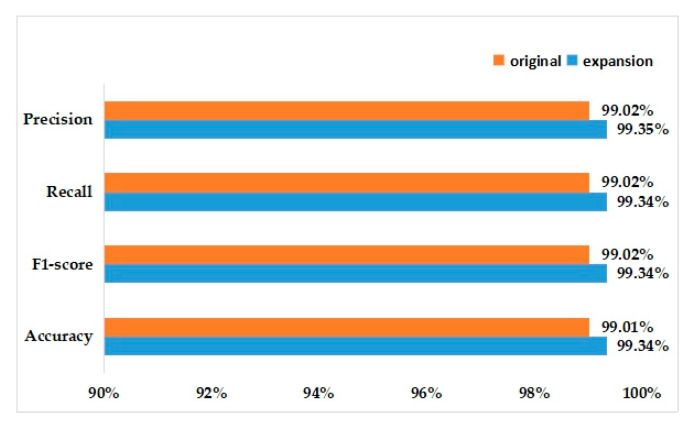
Recognition accuracy for the initial and expanded data.

In the same experimental setup, the model we proposed is trained on the augmented 21,410 images and the final classification accuracy can reach 99.34%. When we used the original dataset to train the model, the accuracy rate can also reach 99.01%. Figure 12 shows the recognition accuracy. It can be seen that after the data expansion, all the measures have been slightly improved on the model proposed.

## 4. Conclusions

An improved convolution neural network model based on VGG16 is proposed in this work. The classifier of classical VGG16 network is modified by adding a batch normalization layer, a global average pooling layer, and a fully connected layer to accelerate convergence and reduce training parameters. The proposed model trains on 2141 apple leaves in the training set to identify apple leaf diseases. The experimental results show that the accuracy of the model test can reach 99.01% after 692 s training. Compared with the classical VGG16 network, the model parameters are reduced by 119,534,592, and the accuracy is improved by 6.3%.

Although the training time is longer than that of AlexNet and ResNet, our model has fewer parameters and a higher accuracy. Compared with GoogleNet and Alex + Inception, some parameters and training time are sacrificed, but our model has the highest accuracy of up to 99.01%. After data expansion, the accuracy of the model can be increased to 99.34%. The convolution neural network proposed in this work can identify apple leaf diseases quickly and accurately and provides a feasible scheme for identifying apple leaf diseases.

In the future, our work can be improved in the following aspects: (1) collecting more kinds and quantities of apple disease pictures to enrich the datasets to train better models, (2) trying other deep convolution neural networks to improve the accuracy and speed of recognition, (3) trying to run other deep learning methods and apply them to the real-time detection of apple diseases.

## Figures and Tables

**Table 1:** Details of the apple leaves.

Classes	Training Number	Test Number
Healthy	1172	168
Scab	360	51
Frogeye Spot	427	60
Cedar rust	182	26
Total	2141	305

**Table 2:** Confusion matrix of the prediction results.

	True Label	Healthy	Scab	Frogeye Spot	Cedar Rust
Predict Label					
Healthy		**167**	1	0	0
Scab		1	**50**	0	0
Frogeye Spot		1	0	**59**	0
Cedar Rust		0	0	0	**26**

**Table 3:** Classification performance comparison among different models.

Classes	Model	Precision	Recall	F1-Score	Accuracy
Healthy	AlexNet	95.27%	95.83%	95.55%	95.83%
	ResNet34	98.14%	94.05%	96.05%	94.05%
	GoogleNet	98.80%	98.21%	98.51%	98.21%
	VGG16	93.14%	97.02%	95.04%	97.02%
	Alex + Inception	98.80%	98.21%	98.51%	98.21%
	**Our Work**	**98.82%**	**99.40%**	**99.11%**	**99.40%**
Scab	AlexNet	90.70%	76.47%	82.98%	76.47%
	ResNet34	84.21%	94.12%	88.89%	94.21%
	GoogleNet	97.96%	94.12%	96.00%	94.12%
	VGG16	90.91%	78.43%	84.21%	78.43%
	Alex + Inception	97.87%	90.20%	93.88%	90.20%
	**Our Work**	**98.04%**	**98.04%**	**98.04%**	**98.04%**
Frogeye Spot	AlexNet	92.06%	96.67%	94.31%	96.67%
	ResNet34	98.36%	**100%**	**99.17%**	96.67%
	GoogleNet	96.77%	**100%**	98.36%	**100%**
	VGG16	94.92%	93.33%	94.12%	93.33%
	Alex + Inception	95.24%	**100%**	97.56%	100%
	**Our Work**	**100%**	98.00%	99.16%	98.33%
Cedar rust	AlexNet	86.67%	**100%**	92.86%	**100%**
	ResNet34	**100%**	**100%**	**100%**	**100%**
	GoogleNet	92.59%	96.15%	94.34%	96.15%
	VGG16	92.59%	96.15%	94.34%	96.15%
	Alex + Inception	89.29%	96.15%	92.59%	96.15%
	**Our Work**	**100%**	**100%**	**100%**	**100%**

**Table 4:** Comparison of the training parameters and time.

Model	Parameter	Training Time (s)
AlexNet	58,297,732	360
ResNet34	22,671,492	685
GoogleNet	5,716,848	604
VGG16	134,252,356	123,007
Alex + Inception	5,654,356	491
Our Work	14,717,764	692
